# The Recovery of China’s Industrial Parks in the First Wave of COVID-19

**DOI:** 10.3390/ijerph192215035

**Published:** 2022-11-15

**Authors:** Changcheng Kan, Qiwei Ma, Zhaoya Gong, Yuanjing Qi, Anrong Dang

**Affiliations:** 1 Baidu.com Times Technology (Beijing) Co., Ltd., Beijing 100085, China; 2 School of Architecture, Tsinghua University, Beijing 100084, China; 3 School of Urban Planning & Design, Peking University Shenzhen Graduate School, Shenzhen 518055, China; 4 Key Laboratory of Earth Surface System and Human-Earth Relations of Ministry of Natural Resources of China, Peking University Shenzhen Graduate School, Shenzhen 518055, China; 5 School of Soil and Water Conservation, Beijing Forestry University, Beijing 100083, China

**Keywords:** industrial parks, production recovery, spatial heterogeneity, COVID-19

## Abstract

Industrial parks are functional urban areas that carry the capacity to support highly concentrated production activities. The robustness and anti-interference ability of these areas are of great importance to maintaining economic vitality of a country. Focusing on the rate of production recovery (RPR), this paper examines the recovery of 436 major industrial parks in mainland China during the first wave of COVID-19. Leveraging spatio-temporal big data, we measured 14 attributes pertaining to industrial parks, covering four categories, namely spatial location, central city, park development, and public service. We focused on the spatial association and heterogeneity of the recovery patterns and identified the factors that truly affected the recovery of industrial parks with quantitative evaluation of their effects. The results reveal that: (1) RPR of industrial parks are significantly spatially clustered, with an obvious “cold spot” in the early outbreak area of Hubei Province and a prominent “center-periphery” pattern in developed areas, which is highly correlated with the spread of the epidemic. (2) The mechanisms driving the resumption of industrial parks are complex and versatile. All four categories in the variable matrix are related to RPR, including up to eight effective influencing factors. The effect of influencing factors is spatially heterogeneous, and its intensity varies significantly across regions. What is more interesting is that some impact factors show positive effects in some industrial parks while inhibiting the recovery in others. On the basis of the discussion of those findings with practical experiences, the planning and construction strategies of industrial park are suggested to mitigate the impact of similar external shocks.

## 1. Introduction

Industrial parks have significant capacities in hosting economic activities [1]. Concentrating on specialized industries, industrial parks can effectively take advantages of agglomeration economy, becoming powerful engines of urban and reginal economic growth. As of October 2019, there were over 15,000 industrial parks of various industries in China mainland, contributing more than 30 percent of the Chinese economy [2]. In Spring Festival 2020, the outbreak of COVID-19 exerted a severe shock on the production of industrial parks and put them under huge risks [3,4,5]. It is of great importance to studying the production recovery of industrial parks under risk, and the influencing factors that targeted supporting policies for recovery can be suggested with the consideration of controlling the pandemic at the same time.

The study on the capacity of socioeconomic entities to recover facing unexpected shocks mainly focuses on three aspects. The first aspect is the scientific assessment on the recovery level and resilience of the economy [6]. Wu et al. [7] evaluated the post-disaster resilience of Louisiana after Hurricane Katrina in 2005 in terms of infrastructure, economy, society, and natural environment. Some studies made more refined assessments from the perspective of spatial-temporal heterogeneity [8,9]. For example, Zhou, Liu, and Fan [10] conducted a study on the efficiency of economic recovery of Wenchuan earthquake-stricken areas in the short, medium, and long term, respectively. The second aspect is the study of the influencing factors of recovery [11]. Jha et al. [12], the Rockefeller Foundation and Arup [13], divided the driving factors into several categories and subcategories, such as regional status, economic structure, and management system. Martin et al. [14] analyzed various factors that related to social vulnerability, including economy, population, and community structure, in Boston’s post-disaster recovery. Thirdly, a number of studies were carried out on the planning strategies for resilient cities and communities [15]. One representative work in this area is the Resilient City Planning Framework, or RCPF, proposed by [16]. The framework takes into account complexity and uncertainty and covers economic, social, spatial, and physical factors, involving a diverse range of stakeholders. The framework consists of four concepts, namely urban vulnerability matrix analysis, uncertainty-oriented planning, urban governance, and prevention. Each of the four concepts can be subdivided into 3–4 components, and each component is linked to a specific key question, providing systematic policy recommendations for resilient urban planning in the areas of risk perception, preparedness, implementation strategies, prevention, and recovery, respectively.

After the outbreak of COVID-19, the recovery of cities in the face of major public health emergencies has become a research hotspot [17]. Studies have shown that the factors contributing to the fight against the pandemic are concentrated on a few specific types. First, the organizational capability and efficiency of the government [18]. A study of 276 prefecture-level cities in China [19] found that the capacity of the governance matters more than the size of the city. Besides, the configuration and management of workforce, financial, and material resources play a key role. Second, the built environment of the city. Diversified factors, including urban form, facilities, land use, and road traffic, have been proved to play a non-negligible part in allocating epidemic prevention-related resources efficiently, reducing localized population concentrations, and supporting the operation of the urban epidemic prevention system [20,21,22]. The third dimension is the means of urban management. Technology-driven policies and measures can increase social engagement and connectivity and help maintain the functions of public health, education, and economic systems, thus effectively underpinning crisis management and enhancing the resilience and well-being of cities and communities [23,24,25].

Despite the systematic and in-depth efforts of previous studies, most of them take cities as the basic research unit. However, the recovery of industrial parks is distinctly different from that of cities, in that they mainly function as centers of highly specialized production activities rather than urban life. The implementation of regulatory policies is usually more efficient and consistent due to the centralized and closed management mode within a limited boundary. Moreover, the recovery of industrial parks cannot rely solely on their own capabilities, but it requires external policies and resources from surrounding regions and cities. For these reasons, the findings of city-based research cannot be directly applied to industrial parks. The lack of targeted research has led to a lack of in-depth knowledge of the rules and insufficient basis for decision making, which seriously limits the risk-resistance and recovery ability of industrial parks as engines of economic development.

With the help of the emerging multi-source spatiotemporal big data technologies [26,27,28], this study delves into the driving factors of industrial park resilience and their pathways of functioning, with a view to filling the above-mentioned research gaps. The study focuses on three main issues: (1) depicting the spatial patterns of recovery of industrial parks; (2) identifying key influencing factors in the recovery from a number of characteristics at different scales (region, city and park); and (3) investigating their global and local impacts from the perspectives of spatial dependence and heterogeneity. To achieve the objectives, this paper took 436 major industrial parks in China mainland as the research object. Specifically, the return proportion of work population in industrial parks was used to represent RPR after the first wave of COVID-19, and its spatial distribution patterns were analyzed from the angle of spatial dependence. Then, with the help of OLS and SLM models, the significance and size of global effects of various factors were examined. Furthermore, the MWGR model was employed to investigate the varying local effects of factors over space. This was followed by an in-depth discussion on the driving mechanisms and their spatial heterogeneity of industrial park resilience. Finally, planning and management countermeasures are suggested to improve the performance of industrial parks in the face of external shocks.

## 2. Data and Methodology

### 2.1. Study Area and Data

#### 2.1.1. Study Area 

**Figure 1:**
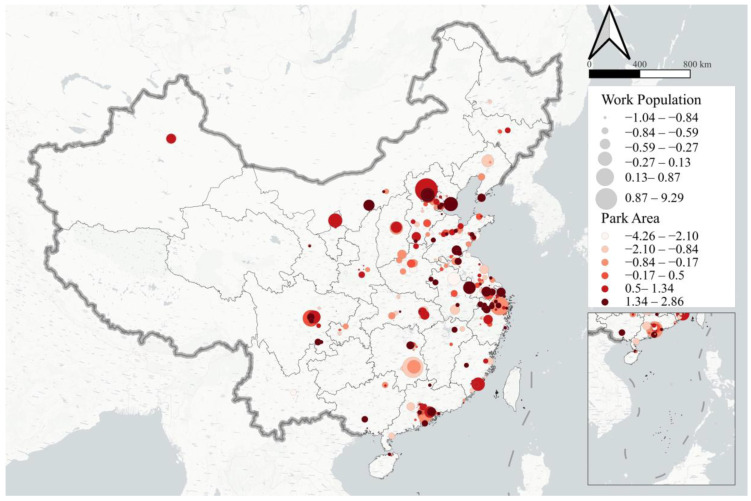
Spatial distribution of industrial parks and basic statistical indexes.

This paper takes industrial parks within China mainland as the object of study, and the boundaries of parks are subject to Baidu Maps. The spatial distribution of industrial parks and basic statistical indexes are shown in Figure 1. In order to keep the analysis robust, the parks with less than 50 hectares of land or less than 10 employees (based on the number of Baidu App users) were excluded, and finally 436 major industrial parks were selected, covering a total area of 911.4 square kilometers and an employment population of 1.5 million people.

#### 2.1.2. Data

(1) Dependent variable: RPR

The return proportion of work population provided by Baidu Maps was used to characterize RPR of industrial parks. The data were collected on 23 March 2020. For a park *i*, the recovery rate $R_{it}$ on the date *t* after the outbreak of the pandemic can be defined as:

$$R_{it}=\frac{P\_\;work_{it}}{P\_\;work_{i}}$$

where $P\_\;work_{i}$ denotes the stablized size of work population in park *i* before the outbreak, and $P\_work_{it}$ is the size of the work population that returned to original jobs in the park on the date *t*.

**Figure 2:**
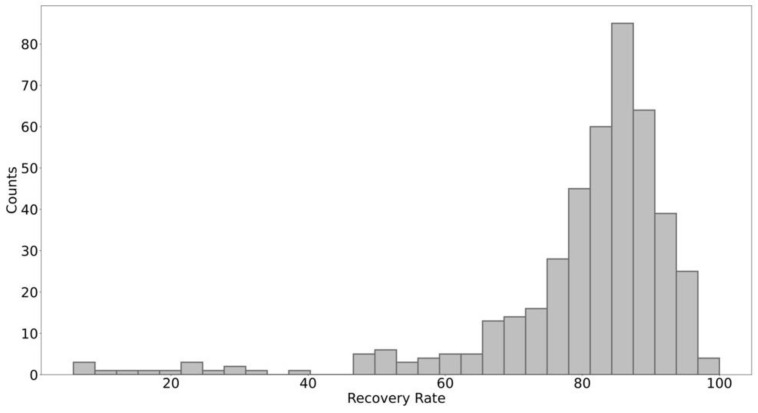
Numerical distribution of recovery rates in industrial parks (The abscissa represents the recovery rate in parks, and the ordinate the number of parks whose recovery rates are in a certain range).

The numerical distribution of RPR is skewed (Figure 2), and mostly concentrated in the range of 82–86%. This indicates that on 23 March 2020, most of the main industrial parks have restored their production organization to some extent, but a considerable number of parks are not promising in terms of recovery.

(2) Independent variables

Many previous works [13,29,30,31] have discussed the relationship between city and community resilience with various factors, including infrastructures, public services, mobility and transportation, management, and so on. Combining the evidence with the authors’ own experience, this paper constructs a variable matrix containing four categories and fourteen factors (Table 1). The four categories include spatial location, central city, park development, and public service, each of which contains several candidate variables. The actual effects of these factors were tested, and effective factors that really influence the recovery of industrial parks were further screened out. All variables in the matrix are logarithmic and normalized to ensure that their effect sizes are comparable.

The spatial location category refers to the adjacency of transportation facilities of each park. Two indexes were selected for measurement. (1) Accessibility of high-speed railway (HSR) (A_hsr). HSRs are one of the main means of labor transport for most industrial parks in mainland China; (2) Accessibility of airport (A_air). It affects the passenger and freight capacity of parks, especially the external contacts at a distance.

The central city category represents the intensity of the intervention effect of the central city to which a park belongs to. Four indexes were selected for measurement: (1) Epidemic intensity of the central city (C_epi), indicating how badly the city has been hit by the pandemic; (2) proximity of the central city (C_loc), closely related to the spatial interaction between central cities and parks; (3) regulation intensity of the central city (C_Regu), i.e., the strength of travel restriction policies in the city where industrial parks are located; and (4) permanent resident population of the central city (C_res), equivalent to the size of the city.

Park development category measures the development conditions and industrial characteristics of industrial parks. Six indexes are selected: (1) vitality under normal state (P_vital), related to the production capacity and organization ability of the park; (2) park area (P_area), to the extent that sufficient land resources can improve the ability of park to optimize the spatial layout of production activities; (3) the work population under normal condition (P_work), reflecting the normal scale of labor force in the park; (4)–(6) are the shares of work population in light industry (LI), machinery manufacturing industry (MMI) and the energy, mining, and chemical industry (EMCI) of the park, respectively, reflecting the industrial structure characteristics.

The public service category represents the service level of living and medical care of each park. Two indexes are formulated: (1) level of medical services (S_med), indicating the park’s ability to diagnose and control diseases, especially response to the pandemic; and (2) level of community services (S_serv), showing the park’s ability to guarantee the livelihood of employees.

### 2.2. Methodology

#### 2.2.1. Spatial Autocorrelation Analysis

Given the similarity of epidemic control and work resumption policies in neighboring cities and regions, spatial autocorrelation analysis was used to characterize the spatial pattern of RPR [34]. The measurement falls into global and local spatial autocorrelation depending on the scale of analysis. The former focuses on the spatial distribution characteristics of a certain geographical attribute in the whole study area, while the latter reflects the degree of association between a local spatial unit and its surrounding areas, also known as local indicators of spatial association (LISA). In this study, *Moran’s I* and *Local Moran’s I* were used to reflect the two types of spatial autocorrelation above [35].

$$Moran\prime s\;I=\frac{n\sum_{i=1}^{n}\sum_{j=1}^{n}(x_{i}-\bar{x})(x_{j}-\bar{x})}{\sum_{i=1}^{n}\sum_{j=1}^{n}W_{ij}\sum_{i=1}^{n}(x_{j}-\bar{x})}$$

$$Local\;Moran\prime s\;I=(\frac{x_{i}-\bar{x}}{m})\sum_{i=1}^{n}W_{ij}(x_{j}-\bar{x})$$

where n denotes the total sample count of industrial parks. $x_{i}$ and $x_{j}$ are the recovery rates of park *i* and park *j*, respectively. $\bar{x}$ is the mean recovery rate in all parks. $W_{ij}$ denotes the spatial weight matrix, and $m=(\sum_{j=1,j\neq i}^{n}x_{j}^{2})/(n-1)-{\bar{x}}^{2}$.

The values of the two indicators both range from −1 to 1. The positive value indicates a significant spatial agglomeration of high/low recovery rate, which are called H-H type and L-L type. However, negative values indicate significant spatial disparities among evaluation units, namely H-L type and L-H type.

#### 2.2.2. Global Factor Impact Analysis: OLS and SLM Models

(1) OLS

In order to quantitatively evaluate the impact of various factors on the resilience of industrial parks, the OLS model was introduced to explore the correlation between the 14 variables (Table 2) and RPR.

$$y=\beta_{0}+\overset{14}{\underset{k=1}{\sum }}\beta_{k}x_{k}+\varepsilon$$

where $\beta_{0}$ denotes the intercept, $x_{k}$ denotes the observed value of the *k*-th factor, $\beta_{k}$ denotes the global regression coefficient of the *k*-th factor, and ε denotes the residual of the model.

(2) SLM

There is a risk of ineffectiveness of the OLS model when spatial autocorrelation exists between variables. In this case, it is necessary to consider spatial dependence to boost the performance of the model. The SLM model can mitigate the spatial bias of OLS by considering the attribute values of adjacent geographical units into the model and constructing the spatial lagged variables.

$$y=\rho Wy+\overset{14}{\underset{k=1}{\sum }}\beta_{k}x_{k}+\varepsilon$$

where Wy denotes the spatial lags of the dependent variable. ρ denotes the spatial autoregressive coefficient, which represents the influence of the dependent variable in the adjacent units on the dependent variable in the focal area.

#### 2.2.3. Local Factor Impact Analysis: MGWR Model

OLS and SLM models estimated the global impact of various factors on the recovery rates. Allowing for the vast geographical area of China and the great differences in the characteristics and levels of economic and social status, the resilience mechanism of industrial parks may be spatially heterogeneous. The MGWR model was employed in this paper to further explore this nonstationarity at the local scale.

Different from the traditional GWR model, in which variables share the same optimal bandwidth, the MGWR model employs a multi-bandwidth method, which allows the bandwidth of different variables to vary, ending up with different spatial smoothing levels. Meanwhile, the bandwidth of each variable is used to characterize its spatial process, which makes the model more credible [36].

$$y=\overset{14}{\underset{k=1}{\sum }}\beta_{bk}\left(u,v\right)x_{k}+\varepsilon$$

where $\beta_{bki}$ denotes the regression coefficient of the local variable, bki denotes the bandwidth corresponding to the regression coefficient of variable *k*, and $\left(u_{i},v_{i}\right)$ denotes the geospatial coordinates of industrial park *i*.

## 3. Results

### 3.1. Spatial Autocorrelation of Recovery Rates 

**Figure 3:**
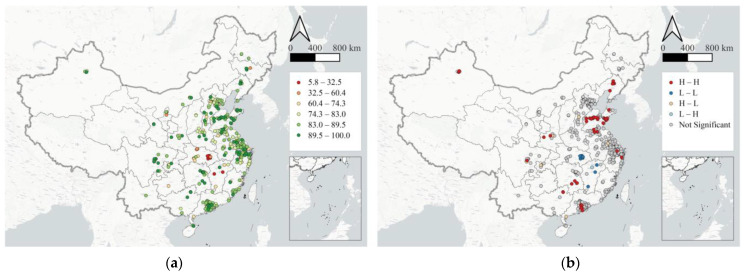
The spatial distribution (**a**) and the LISA indices (**b**) of the average recovery rate of main industrial parks in each city.

Figure 3a shows the spatial distribution of the average RPR for cities in the study area. RPR is distinctly low in Hubei province and surrounding areas. Hubei was the most affected by the outbreak, and they were still under strict restrictions at the time this study was conducted, which means most industrial parks in them are unable to recover successfully. In developed regions such as Beijing, Tianjin, Hebei, Pearl River Delta, and Yangtze River Delta, the spatial distribution of RPR shows the pattern of “low rates in the center and high ones in the periphery”. On the one hand, RPR in Beijing, Shanghai, Guangzhou, Shenzhen, and other core cities mostly range from 60% to 80%, indicating that a considerable number of industrial parks in the above cities have not fully resumed. On the other hand, around these central cities, a significant number of cities feature high levels of recovery, and some have even largely completed the resumption of work. Other regions are generally characterized by the spatial heterogeneity of “imbalanced resumption”. Apart from Qinghai, Gansu, and Tibet, most provinces have some parks basically achieve full resumption of production, becoming the “engine” to boost the regional economy.

The calculated Global *Moran’s I* is 0.418, given the neighborhood setting as a distance-band weights matrix using an adaptive Gaussian kernel, indicating a generally high level of spatial autocorrelation for the recovery rate of all the industrial parks in the study area. In addition, the local indicator of spatial association shows local clusters of high and low rates of recovery, unraveling the variation of degree in spatial autocorrelation of recovery rates across places. Figure 3b shows that H-H and L-L clusters are dominant. With the largest number, H-H clusters are mainly distributed in the eastern coastal regions of mainland China, especially around Liaoning and Shandong Provinces in the northeast, and around Guangdong and Hunan Provinces in the southeast, indicating that industrial parks in these regions recovered the most. L-L clusters are mainly distributed in Hubei province, especially around Wuhan, which was the epicenter of the first wave of COVID-19 and was the most severely impacted. Under the strict travel ban policy, the resumption of these industrial parks was lagging behind. In addition, Guangxi, as a neighboring province of Hubei, has a very low RPR. Besides, a small number of H-L and L-H clusters are scattered in Chongqing municipality and Jiangxi province.

### 3.2. Global Regression Results

The results of OLS model (Table 3) show that there is a significant correlation between some socioeconomic factors and RPR. (1) In the central city category, the epidemic intensity and permanent resident population affects the restoration speed. (2) Among factors in the park development category, the size of work population and the share of work population in MMI are significantly correlated with RPR, while the vitality under normal state also shows some effect at significance level of 0.1. (3) In the public service category, the level of community services is somewhat correlated with park resilience. Moreover, the two indices of spatial location factors are not significant.

Further diagnosis of OLS model results showed that *Moran’s I* of OLS model calculations, LM LAG and LM ERROR obtained by Lagrange Multiplier tests, were highly significant, indicating that the spatial dependence in the dependent variable of RPR poses specification problems for the OLS model and that it should be handled in further analyses. Given the significant Robust LM (lag) index, the SLM model is preferred.

The results of SLM model (Table 4) prove that it is more suitable than the OLS model. The model explains 56.6% of the model variation, and the AIC also decreases. Meanwhile, the results of SLM model revealed unbiased findings compared to those from the OLS model.

(1) Among the two indexes representing the characteristics of spatial location, the airport accessibility has a positive correlation with RPR in parks at 10% significance level.
(2) Similar to the results of OLS model, the two variables of epidemic intensity and permanent resident population in the central city are significant, in which the former inhibits the work resumption in the park, while the latter plays the opposite role. Meantime, the intensity of travel control policy of the central city shows a certain effect of restraining the work resumption.
(3) Among factors of the park development category, the coefficients of three variables are statistically significant. The vitality under normal state is significantly positively correlated at the 10% level, the size of work population in the park is negatively correlated with RPR at the 1% significance, and the share of work population in MMI promotes the work resumption in the park at the 1% significance level.
(4) In the public service category, the level of community services significantly enhances the facilitation of the resumption process in the park at the 5% significance level, while the level of medical services is insignificant.

### 3.3. Spatial Variation of Factor Influence

**Figure 4:**
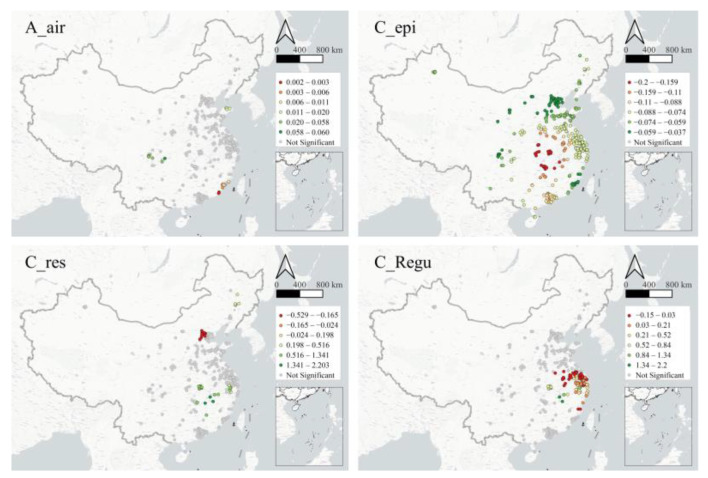

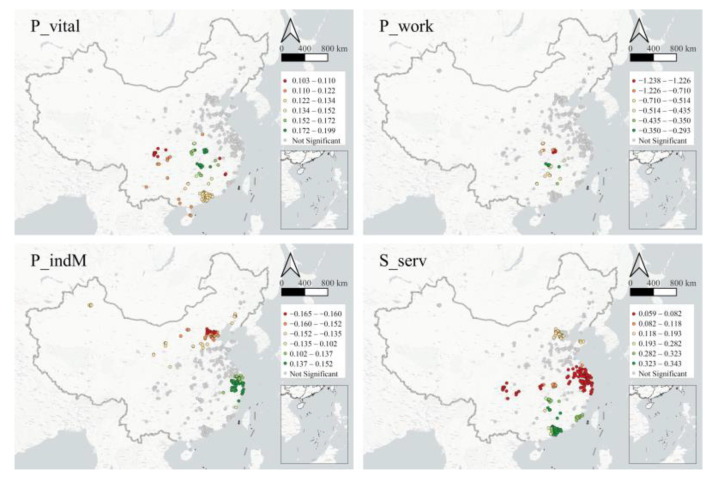
Spatial distribution of the effect sizes of significant factors.

This section further examines the spatial variation of the effects of the eight factors showing global significance in the SLM model, as they are not necessarily significant at the local level. This study shows the coefficients for those samples with statistical significance (Figure 4).

In the spatial location category, (1) the airport accessibility is significantly positively correlated with RPR in Chengdu and Chongqing in the southwestern inland region, Shandong province in the northern coastal region, and Fujian province in the southeastern coastal area. The above areas are commonly featured by remote location and high development level of high-tech industry. Among them, airport accessibility has the strongest positive impact in the southwestern inland region, leaving weak effects in other regions.

In the central city category, (1) the epidemic intensity is the only factor that shows coefficient significance in all parks. The intensity of the inhibitory effect of this factor on RPR in industrial parks is centered in Wuhan, Hubei, and gradually decays outward with the increasing distance. (2) The impact of permanent resident population on park resilience in central cities varies greatly from place to place. In Beijing and Hebei province, the higher the population size of the central cities, the more unfavorable it is for the industrial park to resume production. On the contrary, in the southern provinces of Hubei and Anhui, this index plays a significant role in promoting the restoration. (3) On the whole, the regulation intensity policies in central cities are not conducive to the work resumption, which is more significant in the Yangtze River Delta and Fujian province. On the other hand, in the periphery of Hubei and Anhui provinces, high intensity of regulation favors the resumption of work, indicating the same spatial heterogeneity in the role of this factor, indicating that the effect of this factor also spatially heterogeneous.

In the park development category, (1) the vitality under normal state generally contributes to the park resilience. In Hubei and Hunan provinces, the positive effect is particularly obvious, and in Sichuan, Guangdong, Guangxi, and other regions, the factor also plays a role to some extent. (2) On the contrary, the size of normal work population in the park shows the inhibitory effect on production recovery in different regions, especially in Hubei and Hunan provinces. (3) The effect of the share of work population in MMI has strong spatial disparities. The parks with significant local coefficients are mainly concentrated in the Beijing–Tianjin–Hebei region and the Yangtze River Delta, in which the former is negatively affected by this factor, while in the latter, those parks with a high share of work population in MMI have a significantly high RPR.

In the public service category, (1) the level of community public services plays an effective role in guaranteeing and promoting the resumption of work in parks, covering a wide area. In the Beijing–Tianjin–Hebei region, the Yangtze River Delta region, the Pearl River Delta region, Chengdu–Chongqing region, and central Hubei and Hunan provinces, this factor has played a positive effect, especially in the Pearl River Delta region.

## 4. Discussion

The results of spatial autocorrelation, OLS model, SLM model, and MGWR model show that the recovery of industrial parks is characterized by complex spatial patterns and can be associated with different influencing factors. One or more factors in each of four categories may have a significant impact on the production recovery, indicating that RPR is subject to a synergy effect from diversified factors at the scales of region, city, and park. All significant factors play distinguishing roles in different regions; that is, some factors promote work resumption in some regions while exerting negative effects in other regions.

(1) Spatial location category

As major regional transport facilities, airports are characterized by limited carrying capacity and strong flexibility, which means that air transport is more suitable for industries such as the manufacturing of precision instrument or high-tech electronic equipment with low demand of raw materials and goods and low density of work population. Therefore, they have a strong support for industrial parks with remote locations and a high degree of industrial science and technology. In terms of space, Chengdu and Chongqing, located in the southwestern inland region, are less convenient for transportation, so airports play a more significant role. Focusing on the characteristics of easy management, strong anti-jamming capability, and low sensitivity to distance under abnormal conditions, China has been improving the impact of airports as the powerhouses of surrounding regions and as “lifeline” projects to secure the operation of industrial parks [37].

(2) Central city category

There are complex interactions and counterbalances among the epidemic intensity, the policy response of the government to the pandemic, and the recovery rate. On the one hand, a large number of existing studies [38,39] have shown that social interaction is the main driving factor of the transmission of COVID-19. Therefore, less travel and crowd gathering became the main non-pharmaceutical intervention measures adopted by local governments in mainland China during the first wave of COVID-19 [40]. As a closed and centralized production environment, industrial parks feature higher risks of transmission. As a result, the government tends to impose stronger controls, resulting in slow resumption of work. In terms of the spatial distribution of factors, the epidemic intensity has a universal influence, because China’s epidemic prevention and control policies are highly coordinated across the country, to the extent that many regions are still required to implement certain prevention and control policies despite low risks of outbreak.

Meanwhile, the impact of the pandemic on the work resumption of industrial parks is also moderated by policy control factors. When the epidemic intensity is low, the control measures will directly inhibit the arrival of the work population and the start of normal production [41]. When the epidemic is severe, however, the high intensity of control will help bring the outbreak under control quickly so as to guarantee the gradual recovery of production [42]. The remarkable effect of regulatory policies in the Yangtze River Delta region suggests that emergent controls have a stronger impact on the relatively well-established manufacturing system in the region. In the process of fighting against the epidemic, both central and local governments have begun to recognize the above regulatory role. Therefore, the interaction mechanism between the epidemic intensity and the park resilience during the epidemic prevention and control is now taken into full account to formulate more flexible policies for work resumption [43].

It is worth noting that the park tends to recover faster when there is a central urban area with a high permanent population around it. This may be because the large population size can provide a larger pool of labor force for parks nearby. Concerned by this phenomenon, encouraging the integration of industrial parks and cities has become a fundamental principle in practical urban planning [44]. During the pandemic, cross-regional labor transportation and deployment during the outbreak was extremely impeded, and the supply of local labor has become the most important reliance for the restoration. This mechanism is particularly evident in central China, which was the most affected by the epidemic. On the other hand, the population aggregation in central cities may also lead to an escalation of epidemic prevention and control efforts. In Beijing and its surrounding areas with high safety protection requirements, this potential negative impact is highlighted.

(3) Park development category

The vitality under normal state has a significant positive impact on the resilience. Parks with high production vitality generally feature stable market demand and production capacity, and their mature production organization contributes to their strong ability to resist external risks [45].

The size of the normal work population of the park has a negative impact. The reason is that with the increase of population size, the difficulty of maintaining a reasonable social interaction intensity has increased dramatically, resulting in a relative lack of flexibility in the operation of such parks, making it difficult to adjust production management and advance the resumption process in a timely manner.

From an overall point of view, the share of work population in MMI under normal conditions is conducive to RPR. The possible reason may be that MMI is not labor-intensive, with low density of work population in the production process. In recent years, China has vigorously promoted the informatization and intelligence of manufacturing, which has further reduced the intensity of social contact spatially, so that the risks of epidemic transmission are reduced in MMI enterprises, favoring the resumption of work. The MMI in the Yangtze River Delta is featured by faster upgrading, higher degree of organization, resulting in faster resumption. Contrarily, the development of MMI is relatively out of pace in the Beijing–Tianjin–Hebei region, coupled with stronger regulation, to the extent that it impedes the production recovery.

(4) Public service category

The higher level of community services around the park, the more complete urban life and production service functions of the park, and the higher feasibility of its independent management and operation have thus improved the park’s ability to resist shocks [29,46]. In recent years, the concept of “industrial neighborhood” has been widely valued in the planning of industrial parks. It emphasizes the concept of walkability and proximity to services, and is a logical way to improve the performance of facilities [47].

## 5. Conclusions

Taking advantage of spatiotemporal big data, this paper conducted an in-depth investigation of the spatial patterns of the recovery status of major industrial parks in mainland China and examined 14 factors from four categories exerting significant influences on the resumption of industrial parks. This effort aims to gain insights for the decision support of the planning and management process.

The main findings are as follows: (1) eight factors from categories: spatial location, central city, park development, and public service, have significant impacts on the recovery rate of industrial parks. (2) Different factors have distinguishing effects on recovery rates of industrial parks, featuring a spatial heterogeneity in their influencing effects. These findings suggest that the resilience mechanism of industrial parks can be optimized based on the identification of influencing factors, and the risk resistance and resilience of industrial parks in the face of public health emergencies can be effectively improved by proposing reasonable planning optimization and guidance strategies.

The analysis of model results provides several implications for the improvement of industrial park resilience. (1) Strengthening the “point-to-point” air transport network and promoting the spatial integration of airports and cities. It is required to fully consider the correspondence with industrial parks, coordinate the spatial relationship between airports, cities, and industrial parks, and build the corridors between airports and industrial parks with high standards, leaving the possibility of implementing closed management when necessary. (2) Keeping the epidemic prevention policies more targeted, and appropriately promoting the jobs–housing balance. In order to formulate and implement a more flexible policy of returning to work, the intensity of control should be determined scientifically according to the intensity of epidemic. Economic vitality is maintained with relatively lenient policies in areas with low risk, while safety is the primary prerequisite for resuming work in areas with severe epidemics. On the other hand, to support the mode of industry-city integration, commuting efficiency and the reduction of infection risk during commuting must be guaranteed. Meantime, efforts shall be made to improve the matching of local labor supply and demand. (3) Promoting the maturity of organization and advancement of industrial structure. Industrial parks should focus on the quality of development rather than the size simply and implement the concept of “smart growth” in the construction and operation. On the other hand, to minimize the risks of outbreak arising from crowd gathering, it is necessary to propel the automation and intelligence of industries and the digitalization of park management. (4) Building comprehensive industrial parks with the concept of “industrial neighborhood”. The planning should, firstly, reasonably divide the service areas of industrial neighborhoods according to the walking distance of work population, determine the scale of industrial neighborhoods, allocate service centers, and establish the overall layout structure. The second is to clarify the composition features of various industrial populations and their preferences for public services for the precise allocation of public service resources.

There are limitations in this study which can be further improved in the following two aspects. First, the monitoring data currently used are of a cross-sectional nature and fail to reflect the complete process of resumption. It is planned to extend the time span of the data, investigating the factor effects over time. Second, this study focuses on the domestic factors within mainland China and does not take into account foreign factors that may affect RPR, such as the foreign order demand for protective gears during the pandemic. We leave this for future studies.

## Figures and Tables

**Table 1:** Basic situation of variables.

Category	Influencing Factors	Code	Unit	Description	Data Source	Acquisition Time
Spatial location	Accessibility of high-speed railway	A_hsr	Nr./sq.km	The number of HSR stations within 20 km of the park	Baidu Maps	23 March 2020
	Airport accessibility	A_air	Nr./sq.km	The number of airports within 20 km of the park		
Central city	Epidemic intensity	C_epi	%	The proportion of cumulative number of confirmed cases as of 23 March 2020 in the park-located city to the total urban population	Official statistics released by each city’s CDC	24 January 2020 to 23 March 2020
	Permanent resident population	C_res	Thousand	Total permanent resident population in the city	Baidu Maps	23 March 2020
	Proximity	C_loc	km	The distance from the central point of the industrial park to the government residence of the central city		
	Regulation intensity	C_Regu	times/person	The difference between the number of travel times per capita in the city on 23 March 2019 and 23 March 2020		23 March 2019 and 23 March 2020
Park development	Vitality under normal state	P_vital	-	The production intensity of the park in absence of the pandemic, represented by the average night light intensity of the park in November 2019 [32]	Earth Observation Group	1 November 2019 to 30 November 2019
	Scale of land use	P_area	km^2^	Total area of the park	Baidu Maps	23 March 2020
	Normal work population	P_work	Thousand	The total size of work population in all industries under normal conditions within the park		
	Share of work population in LI	P_indL	%	The proportion of the normal work population in the food processing, textile, and clothing, building materials and home furnishing within the park to the total normal work population		
	Share of work population in MMI	P_indM	%	The proportion of the normal work population in the machinery manufacturing within the park to the total normal work population		
	Share of work population in EMCI	P_indF	%	The proportion of the normal work population in the energy, mining, and chemical industry within the park to the total normal work population		
Public service	Level of medical services	S_med	Nr./sq.km	Per capita access to community service facilities by the work population within a 15-min living circle (1 km) in the park and surrounding areas [33]		
	Level of community services	S_serv	Nr./sq.km	Per capita access to general hospitals within a 15-min living circle (1 km) in the park and surrounding areas		

**Table 2:** Statistical characteristics of variables.

Category	Influencing Factors	Mean	Std	Min	Max
Spatial location	A_hsr	0.71	0.92	0.00	5.00
	A_air	0.22	0.41	0.00	1.00
Central city	C_epi	1.53 × 10^−4^	7.85 × 10^−4^	0.00	4.95 × 10^−3^
	C_res	7526.28	5155.83	542.16	20,370.20
	C_loc				
	C_Regu	0.20	0.08	0.04	0.49
Park development	P_vital	13.45	14.42	0.00	159.80
	P_area	2.10	18.59	0.50	385.40
	P_work	3.38	5.45	0.01	51.35
	P_indL	0.08	0.06	0.00	1.00
	P_indM	0.05	0.05	0.00	1.00
	P_indF	0.05	0.04	0.00	1.00
Public service	S_med	1.46 × 10^−4^	1.25 × 10^−3^	0.00	2.50 × 10^−2^
	S_serv	0.04	0.13	0.00	2.60

**Table 3:** The results of OLS model.

Category	Variable	Coefficient	Std Error	*t*-Value	*p*-Value
Spatial location	A_hsr	−0.023	0.039	−0.583	0.560
	A_air	0.032	0.023	1.395	0.163
Central city	C_epi	−0.635 ***	0.039	−16.370	0.000
	C_res.	0.072 ^†^	0.043	1.665	0.097
	C_loc	0.022	0.039	0.580	0.562
	C_Regu	−0.031	0.027	−1.142	0.253
Park development	P_vital	0.077 ^†^	0.041	1.855	0.064
	P_area	0.026	0.038	0.690	0.491
	P_work	−0.161 ***	0.044	−3.623	0.000
	P_indL	0.017	0.037	0.461	0.645
	P_indM	0.111 **	0.037	2.986	0.003
	P_indF	0.037	0.036	1.049	0.295
Public service	S_med	−0.070	0.059	−1.200	0.230
	S_serv	0.072 ^†^	0.043	1.665	0.097
Adjusted R-squared		0.534954			
AICc		928.496			
*Moran’s I* (error)		4.8224			0.000
Lagrange Multiplier (lag)		13.7655			0.000
Lagrange Multiplier (error)		9.3654			0.000
Robust LM (lag)		4.657			0.031
Robust LM (error)		0.257			0.613

^†^ *p* < 0.1, ** *p* < 0.01, *** *p* < 0.001.

**Table 4:** SLM model results.

Category	Variable	Coefficient	Std Error	t-Value	*p*-Value
Spatial location	A_hsr	−0.020	0.037	−0.537	0.592
	A_air	0.043 ^†^	0.024	1.85	0.064
Central city	C_epi	−0.652 ***	0.038	−17.746	0.000
	C_res	0.030 ^†^	0.019	1.665	0.096
	C_loc	0.022	0.039	0.580	0.562
	C_Regu	−0.053 **	0.021	−2.742	0.006
Park development	P_vital	0.035 ^†^	0.041	1.779	0.075
	P_area	0.028	0.019	0.771	0.441
	P_work	−0.134 **	0.042	−3.166	0.002
	P_indL	0.005	0.003	1.484	0.138
	P_indM	0.123 **	0.036	3.416	0.001
	P_indF	0.036	0.037	0.982	0.236
Public service	S_med	−0.027	0.058	−0.465	0.642
	S_serv	0.075 *	0.038	2.165	0.030
R-squared		0.566			
AICc		905.875			

^†^ *p* < 0.1, * *p* < 0.05, ** *p* < 0.01, *** *p* < 0.001.

## Data Availability

Not applicable.
